# Multi-Axis Force Sensor for Human–Robot Interaction Sensing in a Rehabilitation Robotic Device

**DOI:** 10.3390/s17061294

**Published:** 2017-06-05

**Authors:** Victor Grosu, Svetlana Grosu, Bram Vanderborght, Dirk Lefeber, Carlos Rodriguez-Guerrero

**Affiliations:** MECH Department, Vrije Universiteit Brussel and Flanders Make, Pleinlaan 2, Brussels 1050, Belgium; sgrosu@vub.ac.be (S.G.); bram.vanderborght@vub.ac.be (B.V.); dlefeber@vub.ac.be (D.L.); Carlos.Rodriguez.Guerrero@vub.be (C.R.-G.)

**Keywords:** force sensor, human–robot interaction, exoskeletons, rehabilitation robot

## Abstract

Human–robot interaction sensing is a compulsory feature in modern robotic systems where direct contact or close collaboration is desired. Rehabilitation and assistive robotics are fields where interaction forces are required for both safety and increased control performance of the device with a more comfortable experience for the user. In order to provide an efficient interaction feedback between the user and rehabilitation device, high performance sensing units are demanded. This work introduces a novel design of a multi-axis force sensor dedicated for measuring pelvis interaction forces in a rehabilitation exoskeleton device. The sensor is conceived such that it has different sensitivity characteristics for the three axes of interest having also movable parts in order to allow free rotations and limit crosstalk errors. Integrated sensor electronics make it easy to acquire and process data for a real-time distributed system architecture. Two of the developed sensors are integrated and tested in a complex gait rehabilitation device for safe and compliant control.

## 1. Introduction

The initial meaning of robotic systems was to substitute humans and execute precise operations in repetitive and complex industrial applications. Those robots were performing pick and place, welding, painting, assembling, packing and other tasks for manufacturing. Position based control was sufficient to accomplish most of assignments for industrial applications. Modern robotics have changed over the last several years. With more versatile demands, the robots became more sophisticated. Equipped with force, torque, vision, contact, light, sound and other cognitive sensors, they can learn about the environment and perform a multitude of complex tasks [1,2,3,4,5]. While first robots had to work in strictly enclosed environments, novel ones come to work in close proximity to humans in a collaborative way [6,7]. This involves more severe safety aspects to be considered. One specific category of robotic systems that has as a main concern close human–robot interaction is rehabilitation and assistive robotics. Those devices are intended to augment human strength, improve the quality of life for elderly and regain mobility for people with disorders due to diseases or accidents. The acknowledged need for such devices makes it an important research goal with dozens of prototypes developed around the globe. Even though the number of commercially available devices is limited, research results come to prove that robotic assisted therapies are beneficial for both patients and therapists. The patient can profit of immediate and more intensive treatment while the therapist is exempted to perform heavy burden tasks.

Human walking is a complex mechanism involving multiple Degrees of Freedom (DoF) difficult to mimic by robotic systems. This is why many devices are focusing only to provide power for the most obvious joints involved in human walking as hips, knees and ankles (flexion/extension) trying to impose a straight, sagittal plane walking. The problem rises when the pelvis should be left free, which is adding few more DoFs to the system, while a restricted pelvis will result in an uncomfortable, unnatural and even dangerous walking. The role of sensors in these robotic applications is of considerable importance in detecting interaction and contact forces [8,9,10,11]. Stiff control of angular joint positions is not an acceptable option; instead, a method to assess the human–robot interaction has to be provided [12,13,14], and must make sure that the system does not deliver excessive forces or torques. These safety related issues are often solved by using force/torque sensing units [15], or in addition to that, by using compliant actuators [16]. This work will focus on force/torque sensing in robotic devices designed for lower body rehabilitation. These are exoskeleton systems, worn by subjects with locomotion disorders, that impose repetitive gait pattern motions to human limbs so that the neuromuscular system could relearn walking.

Nowadays, rehabilitation exoskeletons are developed focusing on different DoFs. AutoAmbulator (HealthSouth, Birmingham, Alabama) is using DC actuated DoFs at the hip and knee joints, while Lokomat [17], Lopes [18] and Alex [19] are adding to those a passive ankle to avoid foot drop, which can cause injuries while training over treadmill walking. For an appropriate gait, pattern reconstruction and more complex rehabilitation practice systems as ALTACRO [20], Lopes II [21], Alex III [22] and Corbys [23] are implementing active ankle DoF.

Nevertheless, as shown in related research [24,25,26], an important role in normal gait is played by the human pelvis, which performs three translational and three rotational motions while walking. To keep the systems simple, but, at the same time, to provide an increased degree of comfort for the user, many systems implement passive DoFs at the pelvis level. For instance, Lokomat is using passive vertical movement but restricts horizontal translation, pelvic rotation and obliquity. Alex II is also providing DoFs for the pelvis passively supporting the robotic legs. An observed common practice to gain maximal comfort and natural gait patterns for the user is to combine passive and actuated joints as in Lopes [18] and Corbys [23] but doing it in such a way that the controllability of the whole system becomes more challenging. Solving the pelvis freedom problem using passive elements is obviously a step forward but not sufficient for a fully controllable and unconstrained walking. This being one of the reasons why newer designs of exoskeleton devices tend to implement as much as possible active DoF for the pelvis movement. Actively actuating pelvis by imposing prerecorded trajectories is also not a desired approach due to step variability and synchronization problems. This leads to more complex designs capable of sensing human–robot interaction forces by using various force/torque measurement and estimation methods. Lopes [18] for the two pelvis active DoF is using linear slider potentiometers that measure the spring elongations in the Bowden cable driven series elastic actuator system. A Corbys [23] rehabilitation robotic device is using a 6D force torque unit as a connection interface between the orthoses and the mobile platform. A Pelvic Assist Manipulator (PAM), which is a dedicated pelvis mechanism providing five DoFs with the two three-DoF pneumatic robots [27], is using air pressure sensors on both sides of the pistons.

This paper contribution is to present the design and development of a 3-DoF force sensor, designed to meet the specific, above-mentioned demands for actively assisted pelvic modules used in rehabilitation exoskeletons. The sensor is built to match the ALTACRO, an experimental rehabilitation device with a pelvis control mechanism and two leg orthoses. Counting in total 12 active DoFs, ALTACRO is designed with a strong focus on compliant actuation [28], self sustaining body-weight and balance assistance enhancing natural gait kinematics and dynamics. The developed sensor, located between the orthoses and the body-weight support mechanism, has the role to provide human–robot interaction feedback, appropriate for pelvis control made through the five available actuators.

The work is structured as following: in the second section of this paper, the authors define the requirements that need to be achieved and describe in detail the hardware design of the sensor, explaining its work principles. Section 3 shows how the sensor is integrated and used in the rehabilitation device ALTACRO, which controls the pelvis based on interaction information provided by force sensors presented in this paper. Section 4 describes the parameter identification and preliminary human–exoskeleton interaction experiments. Results and discussions are described in Section 5, followed by the conclusions section.

## 2. Sensor Design

While walking, the human pelvis is performing six DoF movements (three translations and three rotations), which are of relative low amplitudes being neglected in earlier robotic system designs; however, the impact of these movements on overall walking patterns is considerably important. To actuate the pelvis, ALTACRO is using one actuator for lateral, two for vertical and two for sagittal translations. Because the actuators can control independently left and right sides of the body, combined linear motions can also impose rotational movements. To measure the human–robot interaction in an exoskeleton system, the sensor should fulfill specific criteria that are also imposed for described design:

- the sensor should read interaction simultaneously on multiple axes;
- has to be placed close to the interaction location to limit structural influences onto the readings;
- it should be small and lightweight not to add unnecessary mass and inertia to the system;
- it should be strong enough to deal with all interaction forces that go through it; and
- the sensor should have different loading characteristics for the three axes of interest.

**Figure 1:**
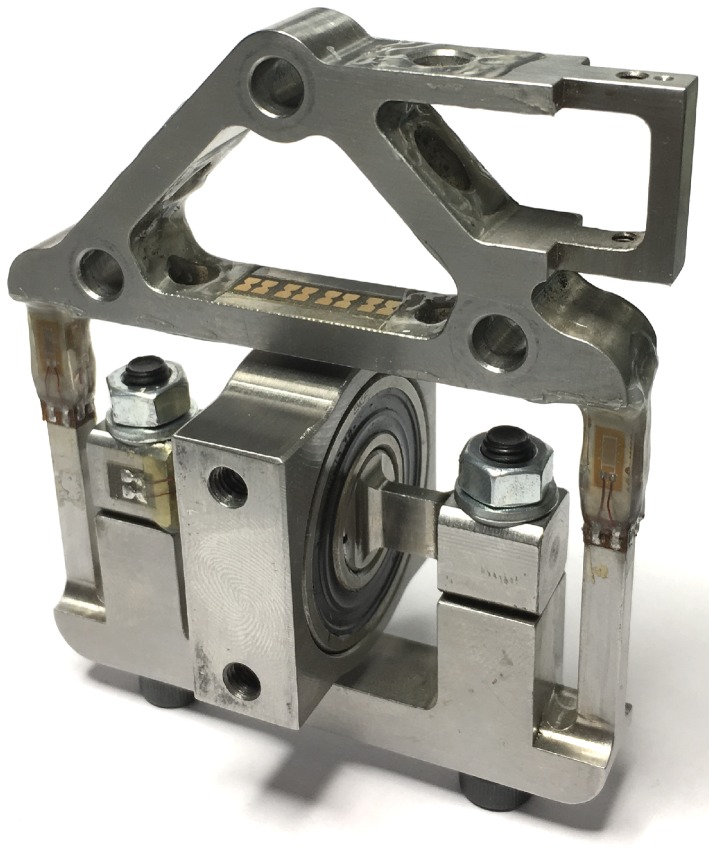
The assembled 3D force sensor with the strain gauges glued to the structure.

**Figure 2:**
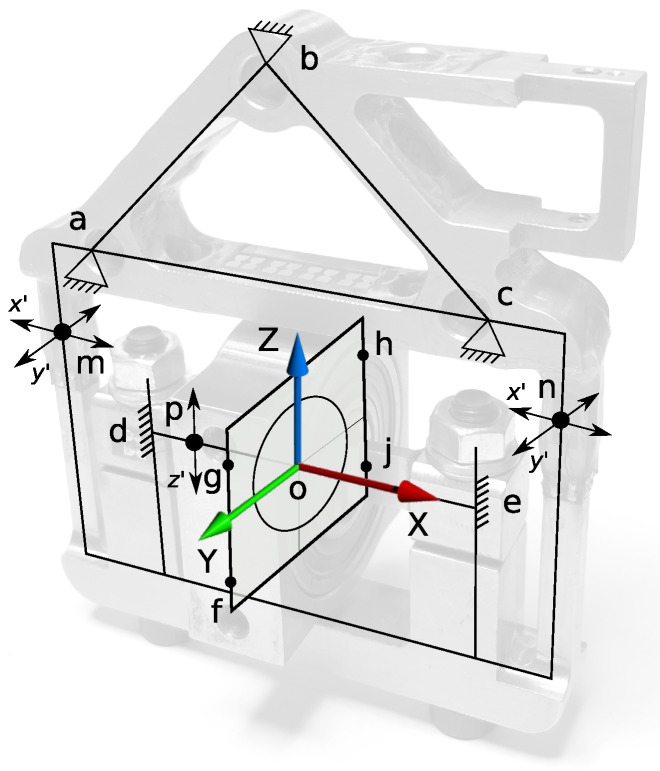
Force sensor structural concept.

**Figure 3:**
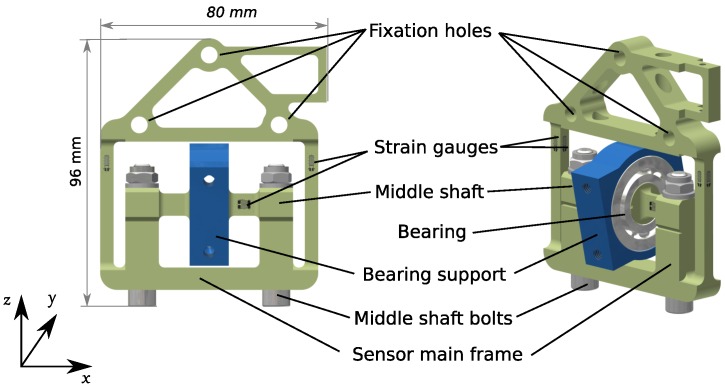
The design consists of a sensor main frame connected to the robot structure via the three fixation holes and supports the orthosis legs by the cantilever beams attached to the bearing support. The bearing is mounted on the middle shaft, which is fixed to the main sensor frame by two fixation bolts. The bearing allows for rotation of ±10 degrees required for hip adduction/abduction.

**Figure 4:**
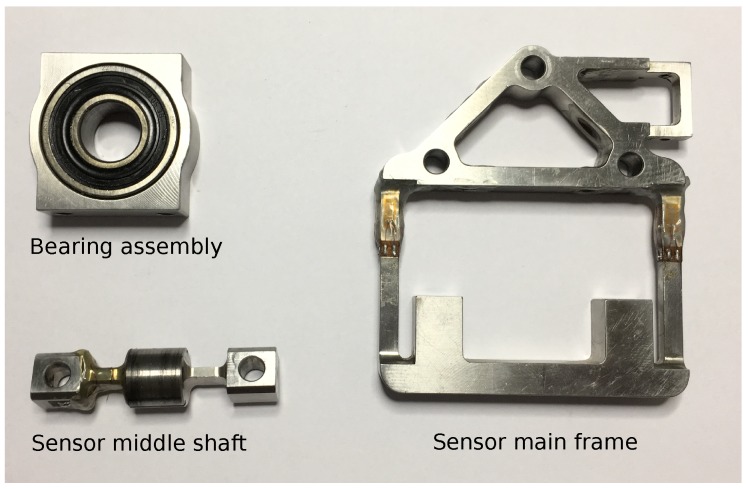
Milled sensor mechanical parts prepared for assembly. Strain gauges are glued to the sensor frame and to the sensor middle shaft.

According to the proposed design concept, we needed to attach the orthosis legs to the support system using a cantilever beam that rotates ±10 degrees around its axis of symmetry for adduction/abduction hip DoF. At the beam end, where it connects to the robotic pelvis, interaction forces with humans have to be measured. Placing a compact 6-DoF commercial force/torque sensor at that location was not possible, and placing it further from the pelvis would overload the bending moment of the sensor and thus also saturate its force readings. The solution was to design a custom, application specific, force sensor presented in Figure 1 with the structural concept shown in Figure 2. The design concept is depicted in Figure 3 and the main parts of the sensor are presented in Figure 4.

In the proposed structural concept (Figure 2), the sensor frame is connected to the main system structure via points *a*, *b* and *c*. Exoskeleton orthosis is attached to the sensor via beams in points *g*, *f* on one side and *h*, *j* on the opposite side of the bearing support block. In this design, instead of supporting the bearing from outside, the inner rings are connected to the main frame using a very short shaft fixed in points *d* and *e*. This leads to a smaller bearing diameter and a high bending stiffness of the cantilever beam.

Another advantage of the design is that all of the forces are measured on the static part of the sensor. The force signals are directly measured in the static frame as required. In contrast, the implementation of a 6-DoF force/torque cell at the end of the cantilever beam would require the online computation of the frame transformation. The approach with the sensing at the static side does not lose any precision due to transformation, but more important is that the design of the multi-axis force sensor can be optimized for anisotropic loads. In the application of an actuated pelvic support, the loads in different orthogonal direction are very distinct. The required load range supported by the sensor, as well as the requirements for sensitivity, are anisotropic. To comply with these, the sensor has to be strong and sensitive for both supporting the heavy weight loads of the human and exoskeleton structure and measure precisely rather small interaction forces that appear in the transverse plane (i.e., weight shifting, balance recovery).

All forces measured by the sensor are exerted via the bearing support block, and its center, noted with *o*, is considered as the origin for the force distribution. The force *x*, which is parallel to the sagital plane (anterior/posterior displacements) is defined by the strain due to bending at the locations *m* and *n*, (*x’*). In addition, *y* forces are measured here due to strains (*y’*) resulted from left/right medio-lateral displacements. The shear strain in point *p*, (*z’*) is used to measure the vertical force *z* (direction of weight, up/down displacements).

The maximum load rating of the multi-axis force sensor is set to 2000 N for the *z*-axis, able to support the full body weight of the user and 300 N for the *x*-axis and *y*-axis, sufficient to support the lateral and sagittal posture of the pelvis. The design requirements for sensitivity is chosen as 1 N for the *x*-axis and *y*-axis to obtain a high transparency of the robot in balancing movements of the pelvis while 5 N are desired for the *z*-axis to obtain a responsive controlled body-weight support. Considering unequal axes loading and sensitivity parameters, a key aspect in the design is to strongly avoid crosstalk induced by the high *z*-load into the sensitive *x* and *y* measurements.

### 2.1. Mechanical Design

To measure forces in the structure designed sensor uses strain gauges. These are electrical resistors that can be bounded on a deformable structure and vary their resistance according to the mechanical strain of the structure. Strain gauges have been chosen mainly for their availability, ease of use and positive experience working with them in other related research projects. Compared, for instance, with piezoelectric sensors, strain gauges achieve better stability for long-term measurements, as well as improved linearity. The disadvantage of the resistive type gauges is the sensitivity to temperature variations, which is solved in this case by building the sensor using full bridge configuration. Capacitive sensors could be another option to consider while those can provide in some cases better performance, but, due to the shelf products not being compatible with the proposed requirements, this solution was not selected. Due to very small strains that can be measured, semiconductor strain gauges were not considered.

#### 2.1.1. *Z*-Axis Design

The *z*-axis acting forces are measured using shear strain gauges applied to the sensor middle shaft, and these are of 062TV type from Micro-Measurements (Wendell, NC, USA). The structure of the middle shaft is kept as short as possible to minimize its bending deformation, and its structure and loading are symmetric such that the shear force equals half of the *z*-force. Currently, only one side of the shaft is populated with the strain gauges but if a higher resolution is required, it is possible to also add strain gauges to the other side. Double sensitivity is obtained if corresponding strain gauges of both sides are wired in series such that a single full bridge is created. The shear measurements on the *z*-axis are not sensitive to the *x*- and *y*-forces. These impose a longitudinal and a bending deformation of the middle shaft, which the shear strain gauge ideally does not register. The shear strain gauges are also placed symmetrically (back-to-back) in a full Wheatstone bridge. If bending due to *y*-forces occurs, the eventual effect in the shear strain gauges on both sides will cancel each other out. The middle shaft is shaped such that its load capability complies with the high force requirement in the *z*-direction, while the strain at the sensing region is optimized for a good sensitivity. The thin rectangular cross-sectional shape maximizes shear deformation, while the bending deformation is kept to a minimum according to the magnitude of anisotropic load.

#### 2.1.2. *X*- and *Y*-Axis Design

The *x*- and *y*-force sensing have a bending beam structure. The square bars in the rigid structure will deform in a S-shape, in the yz-plane as well as in the xz-plane. For a better sensitivity, the strain is measured where the bars have the highest deformation. The sensing Wheatstone bridges are made of simple linear strain gauges of the type 125 BZ from Micro-Measurements. These are symmetrically placed to measure bending strain with all gauges adding equally to the sensitivity. The *x*- and *y*-force sensing are designed to be very insensitive to the high *z*-loads that extend or compress the bars in their axial direction, implying only a limited effect of *x*- and *y*-strain, canceled also due to the configuration of the strain gauges in the sensor.

### 2.2. Data Acquisition Electronics

Before sensor analog signals become understandable to the digital controllers, these have to pass through multiple electronic circuit blocks as for amplification, filtering, and digital conversion.

For a reliable data conversion, many factors should be considered and the electronics have to be designed with special care.

**Figure 5:**
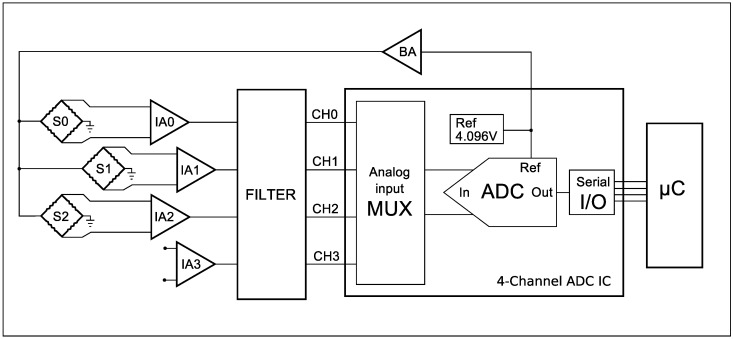
Simplified block diagram of the integrated electronics module with S0, S1 and S2 strain gauges Wheatstone bridges powered by the Buffer Amplifier. Instrumentation amplifiers IA0, IA1 and IA3 provide signals through a filter block to the Analog to Digital Conversion channels CH1, CH2 and CH3. The digital signal can be accessed by the application microcontroller using Serial Peripheral Interface (SPI) communication.

In order to acquire data from the developed sensor, a dedicated electronic module was built. It consists of signal conditioning circuitry with two Precision Dual-Channel Rail-to-Rail instrumentation amplifiers, filters and an on-board Analog to Digital Converter (ADC) Integrated Circuit (IC), as presented in the block diagram of Figure 5.

Low DC excitation voltage was chosen for simplicity, low cost and also to avoid measurement errors resulting from self-heating effects of the current flow in strain gauges. In addition, to avoid significant measurement errors due to long lead-wire resistance, the developed electronic module was attached to the sensor’s frame as close as possible. To provide the sensor with power, the ADC IC on-chip reference voltage of 4.096 V and a buffer amplifier (BA) are used. The signals from the three Wheatstone bridges S0, S1 and S2 are passed, respectively, to the instrumentation amplifiers A0, A1 and A2. The amplification gains of the on-board instrumentation amplifiers can be set individually for each sensor axis using a precision resistor. According to the amplifier IC specification, a maximum gain of 1000 is possible, which is more than sufficient for the proposed application. Before the signals are linked to the 4-channel ADC circuit, they are passed through a low pass filter. The 16-bit resolution ADC IC converts the sensors analog to digital signals with a maximum sample rate of 114 ksps; then, preprocessed data is transmitted by an SPI communication bus to the application node microcontroller (μC), where it is processed into the force value. Considering the high ADC sample rate and the 1 kHz real-time control loop on the application microcontroller, it is possible to acquire additional samples and average them to remove the effect of transition noise on conversion results.

## 3. System Integration

**Figure 6:**
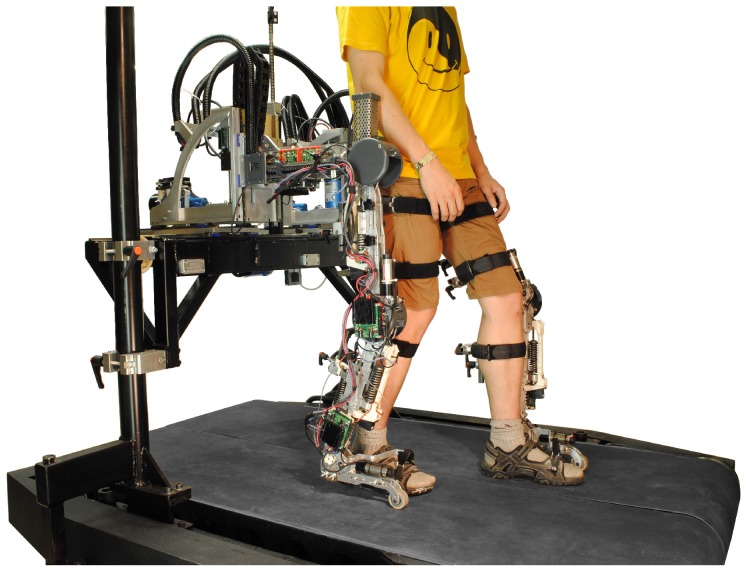
The ALTACRO robot with the orthoses strapped to the person’s legs for over treadmill walking.

The ALTACRO system, presented in Figure 6, has three main structural assemblies. Two slim and light-weight orthoses structures with sliding mechanisms to adapt the exoskeleton size closely to the patient’s legs. The integrated six smart compliant actuators are capable of providing assistive torques for hip, knee and ankle flexion/extension to a human while walking over a treadmill.

**Figure 7:**
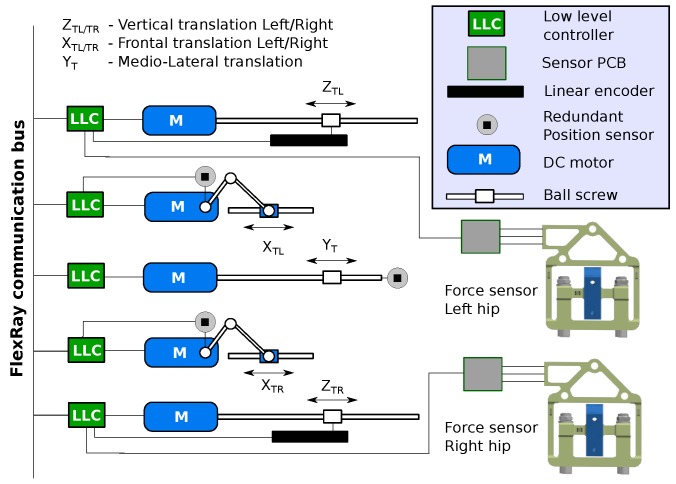
Block diagram of the integrated hardware to control pelvis movements. Two force sensors are installed for the left and right hip sides. In addition, *y*- and *z*-translation motions are made using sliding mechanisms driven by ball screws, while *x* translation motions are made using slider-crank mechanisms. The five actuators are controlled by individual low level control modules that communicate in real time over the FlexRay network bus.

The third part is represented by the support system, which contains five actuated degrees of freedom acting on both exoskeleton legs at the pelvis height level. This sturdy structure can provide full body weight support while also assisting actively in a functional gait pattern using a compliant control algorithm based on feedback from the three-axis force sensors described in this work. The support structure can actuate the lateral and the vertical translation of the pelvis, the internal/external pelvic rotation (rotation in the transverse plane) and pelvic obliquity (rotation in the frontal plane). Two more actuated degrees of freedom are the ad/abduction (rotation in the frontal plane) of the hip for both legs. Currently, the system is operational with ad/abduction rotating freely. A schematic representation of the pelvis support system architecture is presented in Figure 7.

In order to use the sensors for implementing pelvis control algorithms, these have to be integrated in the real-time architecture of the distributed communication and control hardware system. For this purpose, the conditioning electronics of the sensor are connected through the SPI port to the real-time gateways, which communicate over FlexRay protocol to the rest of the system [29]. For platform control and safety reasons, each actuated axis of the support system is equipped with a position sensor. High resolution linear position encoders are used for the vertical motions and for the other pelvis translations, which are obtained from rotational motions, are used incrementally optical in parallel with absolute magnetic encoders. The data from all of the sensors are centralized and processed by the main computing unit that controls the entire system.

## 4. Experiments

### 4.1. Calibration and Parameter Identification

The sensor structure and strain gauges bridges were conceived such that crosstalk effects should be minimal. In practice, the multiple axis sensors always have to deal with the undesired crosstalk effects after fabrication. To define sensor parameters, a series of experiments were performed. The reading electronics of the sensor was separately checked for correct operation using a Strain Indicator Calibrator Vishay Model 1550 A (Wendell, NC, USA). It is a true Wheatstone bridge circuitry able to simulate quarter, half and full bridges for both 120 Ω and 350 Ω gauges with an accuracy of 0.025%. With this experimental setup, we were able to define the Noise, Sensitivity, Gain, Linearity and Full Scale Measurement Range of the amplifier. The parametrized electronic module was connected to the sensor and further experiments were performed using an Instron 5900 test bench (Darmstadt, Germany) for loading the sensor. In order to see how the *z*-axis load is influencing the *x* and *y*-axis measurements, the sensor was installed vertically in the setup using the three fixation holes on one side, and the bearing support holes where the exoskeleton cantilever beams are fixed, on the other side. The sensor was loaded with a force following a sinewave profile with the maximum amplitude of 500 N and 0.05 Hz. To compensate for crosstalk errors, sensor compliance matrices were defined. While a multiple axis load test bench was not available, each sensor was tested using a number of static loads applied independently to the *x*-, *y*- and *z*-axes.

### 4.2. Free Walking

**Figure 8:**
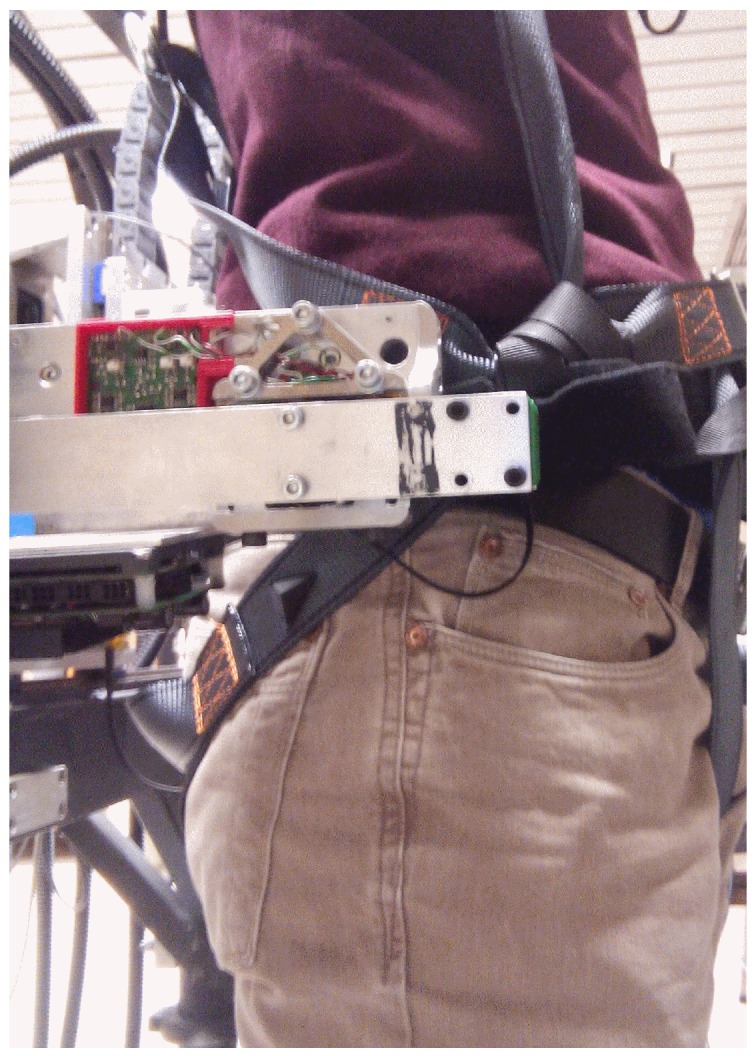
Human pelvis attached to the ALTACRO system from which the leg orthoses have been removed.

Another set of experiments was performed after the sensor parameters were identified, and it was integrated into the ALTACRO system. In order to remove the undesired weight applied to the sensor, robotic orthoses have been dismounted, so a human strapped into the system was able to walk over the treadmill with his/her legs free, as in Figure 8. In this experiment, sensor output data was used to measure human–robot interaction at the pelvis level while walking, and use it as feedback for the admittance control loop of the back platform.

## 5. Results and Discussion

**Figure 9:**
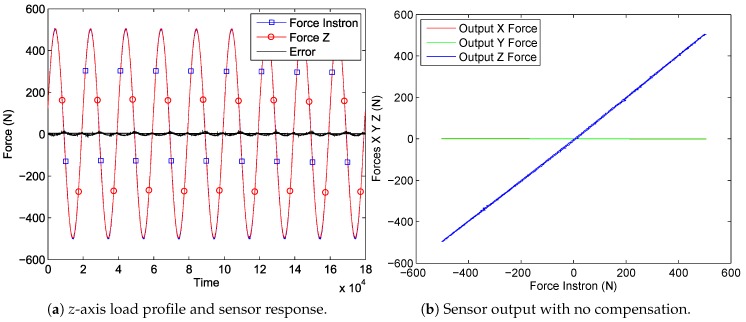
Force sensor tested with an Instron test bench applying a sinusoidal load profile of 500 N amplitude and 0.05 Hz to the sensor’s *z*-axis (the experiment described in Section 4.1).

**Figure 10:**
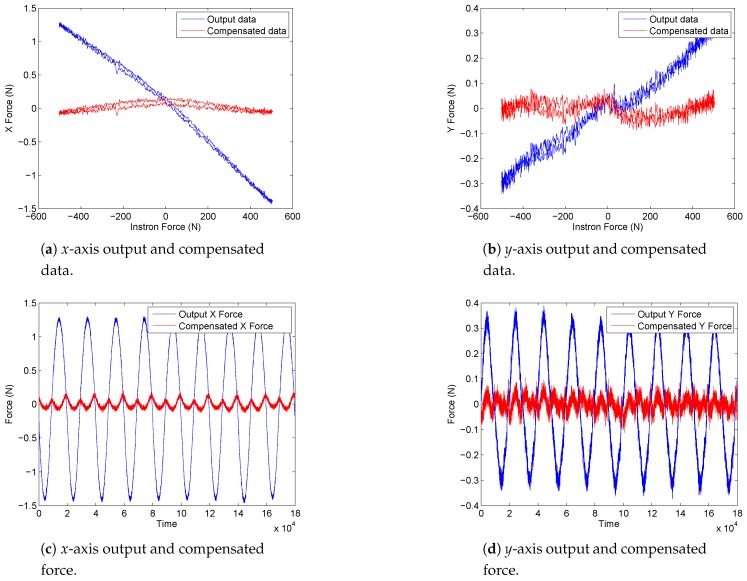
Force sensor response data with and without crosstalk compensation for *x*- and *y*-axes when sinusoidal 500 N load is applied to the *z*-axis (the experiment described in Section 4.1).

The results of the first experiment, described in Section 4.1, are shown in Figure 9a, which represents the loading profile of the Instron device and the sensor response. In Figure 9b, the correlation between the applied force and the measured values from the three axes of the sensor are presented. One can see that the *z* output has a linear correlation to Instron load with only a small error between the two and that *x* and *y* are influenced within a negligible range, at least for this force scale. However, the forces that have to be measured on *x*- and *y*-axes are much smaller, thus a closer look at the data is taken in Figure 10a,b. It is seen that the crosstalk of approximately 1.5 N on the *x*-axis and of 0.3 N on the *y*-axis are induced by the *z*-load of 500 N. Furthermore, in Figure 10a,b, it can be seen that, by applying the crosstalk matrix to the sensor force data, a considerable improvement is obtained with errors below 0.2%. Other functional sensor properties are defined in Table 1.

**Figure 11:**
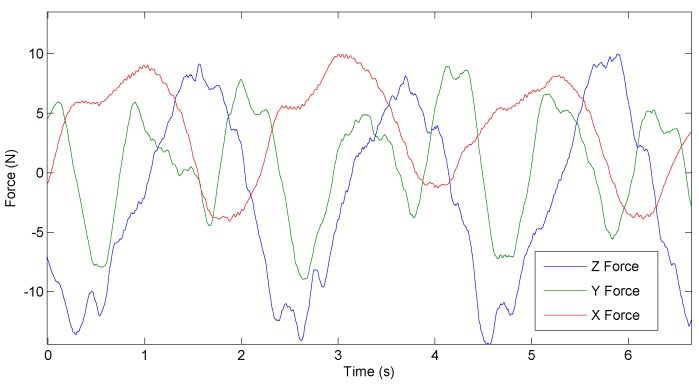
Raw sensor data of the human–robot interaction while human walks with an admittance controlled ALTACRO system on the treadmill with a speed of 1.5 km/h (the experiment described in Section 4.2).

The free walking experiment, described in Section 4.2, was performed to prove that the developed force sensor can be used in a rehabilitation device to estimate human-robot interaction forces. The data in Figure 11 represents *x*, *y* and *z* interaction forces between the user and the robot pelvis modules, while the human walks with the ALTACRO system on the treadmill with a speed of 1.5 km/h.

In this setup, the robotic exoskeleton system is admittance controlled implementing a virtual spring with a stiffness of 1000 N/m between the human body and robotic system. Therefore, the whole behavior of the robot relies primarily on the force feedback quality, which comes from the sensors presented in this article. Acquired data shows that the sensor is very sensitive. It is detecting small interaction forces that are created due to small vibrations in mechanical transmission gearboxes and ball screw mechanisms, but those can be eliminated in hardware and software by applying filtering. Considering that, for this experiment, a healthy subject was walking on the treadmill and the system had to follow the human, interaction forces are relatively small. It can be expected that, by the moment when body weight support and balance will have to be provided, those forces will be considerably higher. Testing sensor behavior in real rehabilitation conditions is an objective for future work, this paper being more focused on presenting sensor design and preliminary experiments.

## 6. Conclusions

The sensor design requirements were successfully accomplished, and the sensor was tested, mounted in the mechanical structure of the robotic rehabilitation device, connected to the real-time network and evaluated on a real scenario, achieving satisfactory results. It does not add to the system unnecessary mass and inertia the sensor being small (80 × 96 mm) and lightweight (340 g). It is integrated in very close proximity to the attachment point with the human pelvis and does detect interaction forces on three axes simultaneously with a good sensitivity. By design, it was possible to obtain different loading characteristics required for weight shifting, balance recovery and gravity compensation forces. The unique qualities of the sensors allows for exploring a whole new set of control strategies that allow the system to act as an active body weight support for patients with sufficient trunk stability. The development of this sensor fulfills the specific requirements for active pelvic modules, which are becoming a trend in modern rehabilitation robotics.

## Figures and Tables

**Table 1:** Identified sensor properties for the *x*-, *y*- and *z*-axes.

Property	X	Y	Z	Units
Full Scale Measurement Range	60	60	2000	N
Maximum load	300	300	2000	N
Sensitivity	18.38	22.32	1.6	mV/N
Resolution	0.05	0.05	0.5	N
Linearity	0.2	0.28	6	N
Hysteresis	-	-	0.4	%
